# Surface characterization, electrochemical properties and in vitro biological properties of Zn-deposited TiO_2_ nanotube surfaces

**DOI:** 10.1038/s41598-023-38733-2

**Published:** 2023-07-14

**Authors:** Salih Durdu, Gizem Cihan, Emine Yalcin, Kultigin Cavusoglu, Atilgan Altinkok, Hasan Sagcan, İlknur Yurtsever, Metin Usta

**Affiliations:** 1 Industrial Engineering, Faculty of Engineering, Giresun University, Merkez, 28200 Giresun, Turkey; 2 Mechanical Engineering, Giresun University, 28200 Giresun, Turkey; 3 Department of Biology, Giresun University, 28200 Giresun, Turkey; 4 Turkish Naval Academy, National Defence University, 34940 Istanbul, Turkey; 5 Department of Medical Laboratory Techniques, Istanbul Medipol University, Istanbul, Turkey; 6 Pharmacology and Toxicology Department, Boonshoft School of Medicine Ohio, Wright State University, Dayton, USA; 7 Materials Science and Engineering, Gebze Technical University, 41400 Gebze, Turkey; 8 Aluminum Research Center (GTU-AAUM), Gebze Technical University, 41400 Gebze, Turkey

**Keywords:** Biomaterials, Biotechnology

## Abstract

In this work, to improve antibacterial, biocompatible and bioactive properties of commercial pure titanium (cp-Ti) for implant applications, the Zn-deposited nanotube surfaces were fabricated on cp-Ti by using combined anodic oxidation (AO) and physical vapor deposition (PVD-TE) methods. Homogenous elemental distributions were observed through all surfaces. Moreover, Zn-deposited surfaces exhibited hydrophobic character while bare Ti surfaces were hydrophilic. Due to the biodegradable behavior of Zn on the nanotube surface, Zn-deposited nanotube surfaces showed higher corrosion current density than bare cp-Ti surface in SBF conditions as expected. In vitro biological properties such as cell viability, ALP activity, protein adsorption, hemolytic activity and antibacterial activity for Gram-positive and Gram-negative bacteria of all surfaces were investigated in detail. Cell viability, ALP activity and antibacterial properties of Zn-deposited nanotube surfaces were significantly improved with respect to bare cp-Ti. Moreover, hemolytic activity and protein adsorption of Zn-deposited nanotube surfaces were decreased. According to these results; a bioactive, biocompatible and antibacterial Zn-deposited nanotube surfaces produced on cp-Ti by using combined AO and PVD techniques can have potential for orthopedic and dental implant applications.

## Introduction

Titanium and its alloys are widely preferred as dental and orthopedic implant applications due to their superior properties, such as low elastic modulus (respect to 316L stainless steel and CoCr alloys), a high strength-to-weight ratio, excellent corrosion resistance and biocompatibility^1–4^. Biocompatible Ti-based implant materials improve health and leads to increased life expectancy are artificial products. However, the mainly disadvantages of implant materials are determined by mode of production, various physical and chemical properties and the nature of the materials^5^. Ti and its alloys exhibit biocompatibility due to the formation of passive oxide film nanolayer^6^. However, Ti-based implants can be lost at post-surgical operations due to insufficient native tissue integration (bioinertless) and possible bacterial colonization in body conditions^7,8^. Therefore, an ideal implant material must indicate both excellent bioactivity (osteogenic activity), bocompatibility and antibacterial ability. To improve bioactivity, biocompatibility and antibacterial ability on titanium surfaces, several coating techniques such as electro deposition (ED)^9^, sol–gel^10^, ion-exchange^11^, magnetron sputtering^12^, micro arc oxidation (MAO)^13,14^ and anodic oxidation (AO)^15–17^ are applied onto Ti-based implant surfaces. Among these methods; the AO is environmentally friendly methods for fabricating oxide nanotube arrays on complex shaped materials^18^. Numerous well-ordered TiO_2_ nanotube arrays can be fabricated on Ti surfaces by altering the AO parameters such as current, voltage, time, electrolyte composition etc.^19,20^.

TiO_2_ nanotube arrays on Ti-based surfaces are widely investigated for different biomedical applications such as drug delivery, biosensors, cardiovascular stents, dental implants and orthopedic implants^21–23^. Osteoinductive potential of TiO_2_ nanotube arrays surfaces was improved compared to smooth surfaces. Moreover, bioactivity of these surfaces enhances the adsorption of extracellular proteins such as fibronectin, fibrinogen and vitronectin, which is an essential factor in the early stage of biomaterials implantation^24^. However, these surfaces can be exposed to bacterial colonization under body condition. This can be resulted in losing of implant materials. To prevent bacterial attachment and proliferation on Ti-based surfaces, antibacterial elements such as silver (Ag)^25–29^, copper (Cu)^9,30,31^ and zinc (Zn)^32–34^ can be doped.

Ag and Cu can indicate cytotoxic effects although Ag and Cu possess much better antimicrobial activity than other antibacterial elements against to Gram positive and Gram negative bacteria^35^. Zn can indicate a relatively weak antibacterial activity compared to Ag and Cu. However, Zn is an important trace element in bone tissue possesses low cytotoxic effect^36^. Furthermore, Zn positively influences the bone mineralization and growth. Moreover, its deprivation impairs the bone metabolism^37,38^. In addition, Zn plays many important roles such as DNA synthesis, nucleic acid metabolism, enzyme activity, biomineralization and hormonal activity^39,40^. Thus, Zn has been of aroused interest of the biomedical community at last years.

Several investigations were carried out on Zn-doped, Zn-incorporated or ZnO nanoparticles deposited TiO_2_ nanotube on titanium and its alloys at last decade for biomedical applications^17,41–46^. Aydin et al. investigated antibacterial and corrosion properties of TiO_2_ nanotube electrodes on pure titanium and they modified TiO_2_ nanotubes with Ag and ZnO nanorod in SBF^41^. Vranceanu et al. evaluated electrochemical properties of Zn-doped hydroxyapatite deposited on TiO_2_ nanotubes on commercial pure titanium by AO and ED processes^42^. Xiang et al. examined folic acid/ZnO quantum dots sealed TiO_2_ nanotubes on commercial pure titanium^43^. Khudhair et al. investigated copper, zinc, and strontium-doped TiO_2_ nanotubes on commercial pure titanium for neural interface applications^17^. Huo et al. examined osteogenic activity and antibacterial effects Zn-incorporated TiO_2_ nanotube arrays on pure titanium^44^. Zhang et al. evaluated osteoinductivity and self-antibacterial activity of Sr/ZnO doped TiO_2_ nanotubes on commercial pure titanium^45^. Roguska et al. antibacterial properties of ZnO and Ag nanoparticle-loaded TiO_2_ nanotubes on pure titanium^46^. However, according to above cited works, homogeneous and high purity surfaces could not be fabricated on surfaces by the nature of coating processes. Thus, it is essential that homogeneous nano-layer surfaces are produced on metal- or ceramic-based surfaces without any impurity under vacuum conditions by PVD-TE methods in this work.

There is no any report on fabrication, characterization, corrosion and in vitro biological properties (bioactivity, biocompatibility and antibacterial properties) of Zn-nanolayer deposited TiO_2_ nanotubes on commercial pure titanium by using combined AO and PVD-TE processes in the literature. In this work, homogeneous and high purity ZnO-based nanotube arrays on cp-Ti surfaces were fabricated by AO and PVD-TE processes for the first time in literature. The present article deals with commercially pure titanium for dental applications, then coated with ZnO-deposited nanotube layers by AO and PVD-TE processes. Initially, nanotube arrays were formed on cp-Ti surfaces by AO process. And then, Zn nanolayers with thickness of 1 nm, 3 nm and 5 nm were deposited on nanotube arrays by PVD-TE process. The surface morphology, elemental distribution, elemental amount, phase structure, topography and wettability of all surfaces were characterized by SEM, EDX-mapping, EDX-area, XRD, AFM and contact angle measurement, respectively. Electrochemical corrosion properties were evaluated under SBF conditions by Tafel extrapolation method. Bacterial adhesions were investigated via Gram-positive such as *Streptococcus pyogenes*,* Staphylococcus aureus*,* Bacillus subtilis* and Gram-negative such as *Pseudomonas aeruginosa*,* Escherichia coli*,* Salmonella typhimurium* microorganisms. In vitro biocompatibility tests such as cytotoxicity, artificial blood adhesion, ALP activity and hemolytic activity were investigated.

## Experimental details

### Sample preparation

Cp-Ti sheets (ASTM F67 Grade 2; Shaanxi Aone Titanium) were cut the sizes of 10 mm × 10 mm × 1 mm and they were ground up to 2000 # SiC sandpapers as given in Table 1. Then, all polished sheets were cleaned in an ultrasonic bath and were dried by heat gun.

**Table 1:** Chemical composition of cp-Ti (wt%).

Substrate	H	O	N	C	Fe	Al	V	Ti
cp-Ti (Grade-2)	0.015	0.25	0.03	0.08	0.3	–	–	Balance

### Anodic oxidation (AO) method

Cp-Ti sheets were coated in NH_4_F (0.5% wt.) and deionized water (5.0% vol.) containing ethylene glycol electrolyte below 30 °C with a DC power supply (GW Instek PSU 400) by AO process. The AO process of all sheets was carried out at 50 V for 1 h. During AO, the cp-Ti and Pt sheets were used as anode and cathode, respectively. At post-treatment AO, the heat treatment was applied to AO surfaces at 450 °C for 1 h. Thus, amorphous phases were transformed to crystalline form^47,48^.

### Physical vapor deposition (PVD-TE) method

**Figure 1:**
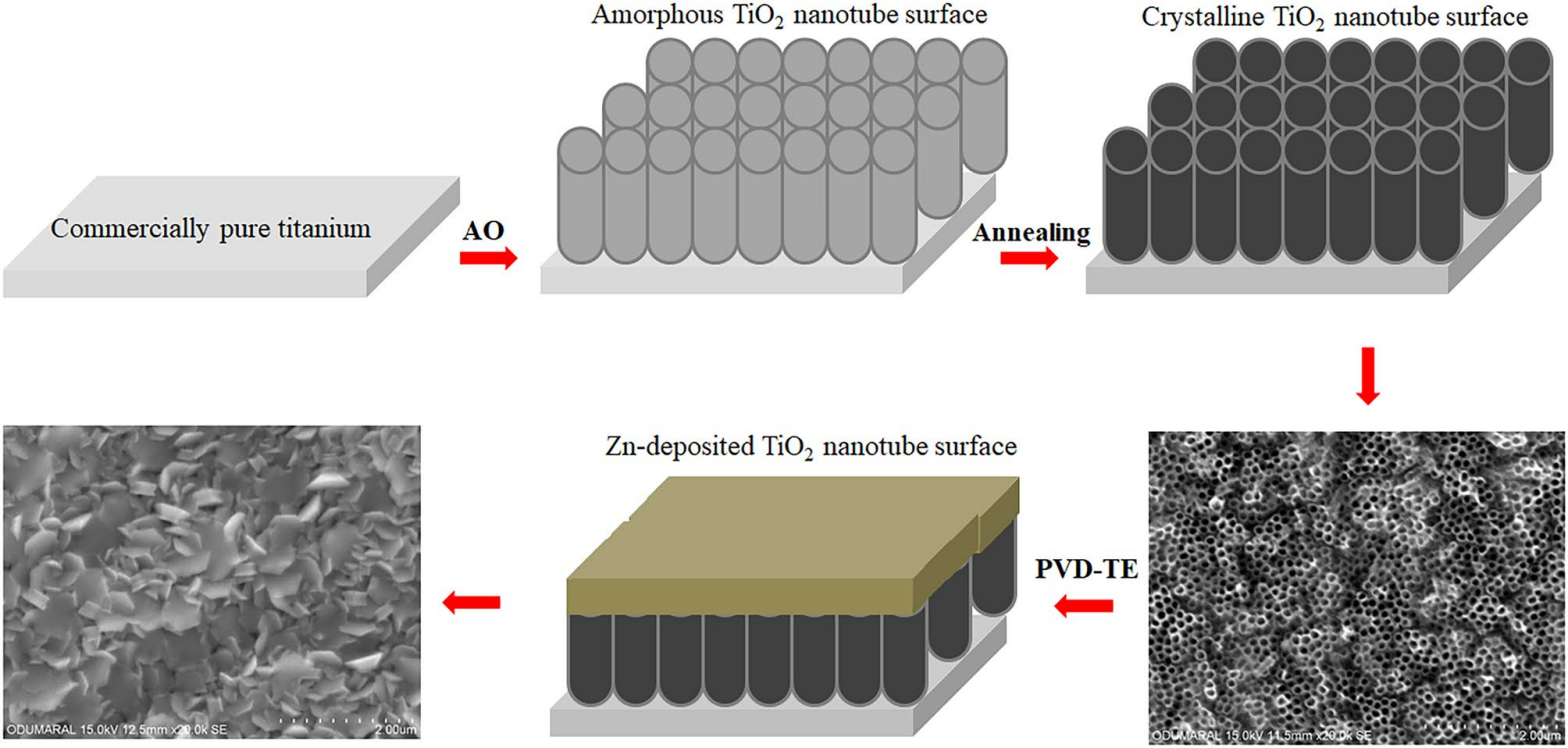
Schematic representation of fabrication of ZnO-based TiO_2_ nanotube arrays on cp-Ti by AO and PVD-TE processes.

The Zn nano layers with 1 nm (Zn-1 nm), 3 nm (Zn-3 nm) and 5 nm (Zn-5 nm) were accumulated onto nanotube arrays by PVD-TE (Vaksis PVD-2T). The vacuum chamber was pumped down until the base pressure reached approximately 1 × 10^–6^ mbar. Chamber pressure increased up to 2 × 10^–5^ mbar owing to the evaporation of zinc powders during process. The Zn was evaporated by increasing the current gradually up to 30 A. The deposition rate was kept as 0.5 A°/s through PVD-TE process. The deposition time varies from 5 to 15 min for all coatings. Wolfram melting pot was used for Zn powders evaporation and this pot was placed approximately 18 cm away from nanotube surfaces. The film thickness and deposition rate were verified by a crystal thickness monitor (CTM) attached to PVD-TE by PVD-2T v2.0 software. CTM and the nanotube surfaces were identical levels in chamber. At the end of process, Zn vapor products were deposited onto nanotube surfaces. Schematic representation of fabrication of ZnO-based TiO_2_ nanotube arrays on cp-Ti by AO and PVD-TE processes were illustrated in Fig. 1.

### Characterization of the surfaces

The morphologies of nanotube and Zn-deposited nanotube surfaces were investigated at 15 kV up to 20.000× by SEM (Hitachi SU 1510). The elemental amounts and distribution of all surfaces were evaluated at 15 kV by EDX- (area and mapping) attached to SEM. Phase structures of the surfaces were analyzed with Cu-Kα irradiation using an X-ray diffraction apparatus (Bruker D8 Advance). Phase determination was performed with an X-ray detector from 20° to 80° at a scanning speed of 1°/min. The surface topography and roughness were investigated in the area of 2.5 µm × 2.5 µm with using a cantilever (TAP-190) by AFM (Nanosurf C3000). The contact angle values of all surfaces were evaluated within 1 min at post-contacted the water droplets of 1 µL with a sessile drop method by contact angle goniometer (Dataphysics OCA-15EC).

### Electrochemical test

Electrochemical tests of the surfaces were performed at 36.5 °C in SBF (simulated body fluid) electrolyte^49^ using Tafel extrapolation method by potentiostat/galvanostat (Metrohm Autolab PGSTAT101). Initially, to determine ideal Tafel potential values, the open circuit potentials (OCP) of the surfaces were measured through 10 min. Then, Tafel extrapolation methods were applied at scanning speed of 1 mV/s between -500 mV and 500 mV of OCP. Three-electrode cells were conventionally used for this test. For electrochemical tests as described previous work^50^, the coated samples, Ag/AgCl and Pt sheet were served as working, counter and references electrodes, respectively.

### Bacterial adhesion test

The antibacterial activity of the coating surfaces was determined by investigating the adhesion of Gram-positive and Gram-negative bacteria. 100 µL of a bacterial culture (0.5 McFarland) was added to the broth medium and 0.5 cm^2^ of coating area was transferred to the medium containing the bacteria. After 24 h of incubation at 37 °C, all coatings were washed twice in succession with 15 mL saline solution. Ultrasonic treatment was performed to obtain the bacteria adhering to the surface. 2 mL of physiological solution was applied to the coatings and sonicated at a frequency of 35 kHz for 5 min. After sonication, 25 µL of the sample was cultivated in nutrient agar dishes to ensure re-cultivation, and colonies were counted after incubation.

### Cell viability test

The cytotoxic effects of each coatings were determined by MTT assay using lymphoblastoid cells (RPMI 8866 cell line). For cell proliferation a medium containing RPMI 1640, penicillin (100 IU/mL), fetal bovine serum (10%), glutamine (2 mM) and streptomycin (100 µg/mL) was used. 0.5 cm^2^ coating was transferred to cell culture containing 2 × 10^5^ cells/mL. The coatings and the cells were incubated for 48 h. Then 5 µg/mL MTT (20 µL) and isopropanol (200 µL) were added to meium. The change in cell viability was determined by spectrophotometric measurement (570 nm) and after 4 h of incubation, the absorbance of the medium was measured.

### Alkaline phosphatase activity

For determination of activity ALP, SAOS-2 cells (primary osteogenic sarcoma) were incubated for 72 h in medium containing 15% fetal calf serum, McCoy's 5A, penicillin, and streptomycin. 0.5 cm^2^ coatings and the cells were from the incubation medium (2 × 10^5^ cells/mL) were interacted for 48 h. At the end of the period, cells were washed with lysis buffer. The solution was centrifuged at 5000 rpm for 10 min after incubation in medium containing MgCl_2_ and Tris (pH 7.6). ALP was analysed with 50 µL of the supernatant, and measurements were made with a spectrophotometer at 410 nm.

### Artificial blood protein adhesion

To determine the hemocompatibility of the coatings, the adhesion of the proteins gamma-globulin, albumin, and fibrinogen was studied. Solutions of blood proteins were prepared at a concentration of 20 mg/mL, and the coating surfaces were incubated with the protein solutions in a batch system for 2 h at 37 °C. After the interaction, a gentle wash with 15 mL of distilled water was performed to remove non-adherent proteins from the surface. The proteins that adhered to the surface were obtained using ultrasonication at a frequency of 35 kHz. The amount of protein separated from the surface after ultrasonication was determined by spectrophotometric measurement (280 nm).

### Hemolytic activity (blood compatibility) test

Hemolytic activity test was applied by the ethical guidelines of the 1975 Declaration of Helsinki. Test procedure was approved by the Ethics Committee of the İstanbul Medipol University of Turkiye (E-10840098-772.02-2348). Patients were informed and consent was obtained for the use of all samples. Blood samples were collected from volunteers after written informed consent was obtained. The hemolytic activity test was performed as described in previous studies with minor modifications^51,52^. Blood taken from volunteer donors was centrifuged at 3000 rpm for 5 min. The resulting cells were washed 3 times with phosphate buffered saline and centrifuged again for 5 min at 3000 rpm. A suspension of erythrocytes diluted in PBS at pH 7.4 was prepared. 600 mL suspensions containing 5 × 108 erythrocytes and coatings (1 cm × 1 cm) were interacted at 37 °C for 8, 16, and 24 h. The cell suspensions were centrifuged at 3000 rpm for 5 min at the end of incubation and the absorbance of the supernatant was measured at 576 nm with an ELISA reader. 1% Triton X-100 was used as positive control, while buffer solution was preferred as negative control. RBC lysis (%) was calculated using the formula below.

$$\% \,{\text{Lysis }} = \, \left( {{\text{A}}_{{{\text{sample}}}} /{\text{A}}_{{{\text{positive}}\,{\text{control}}}} } \right) \, \times { 1}00.$$

### Statistical analysis

Statistical analysis was performed using “IBM SPSS Statistics 22 SP” and all results are given as mean ± SD. p < 0.05 was considered statistically significant.

## Results and discussion

### SEM analysis of the surfaces

**Figure 2:**
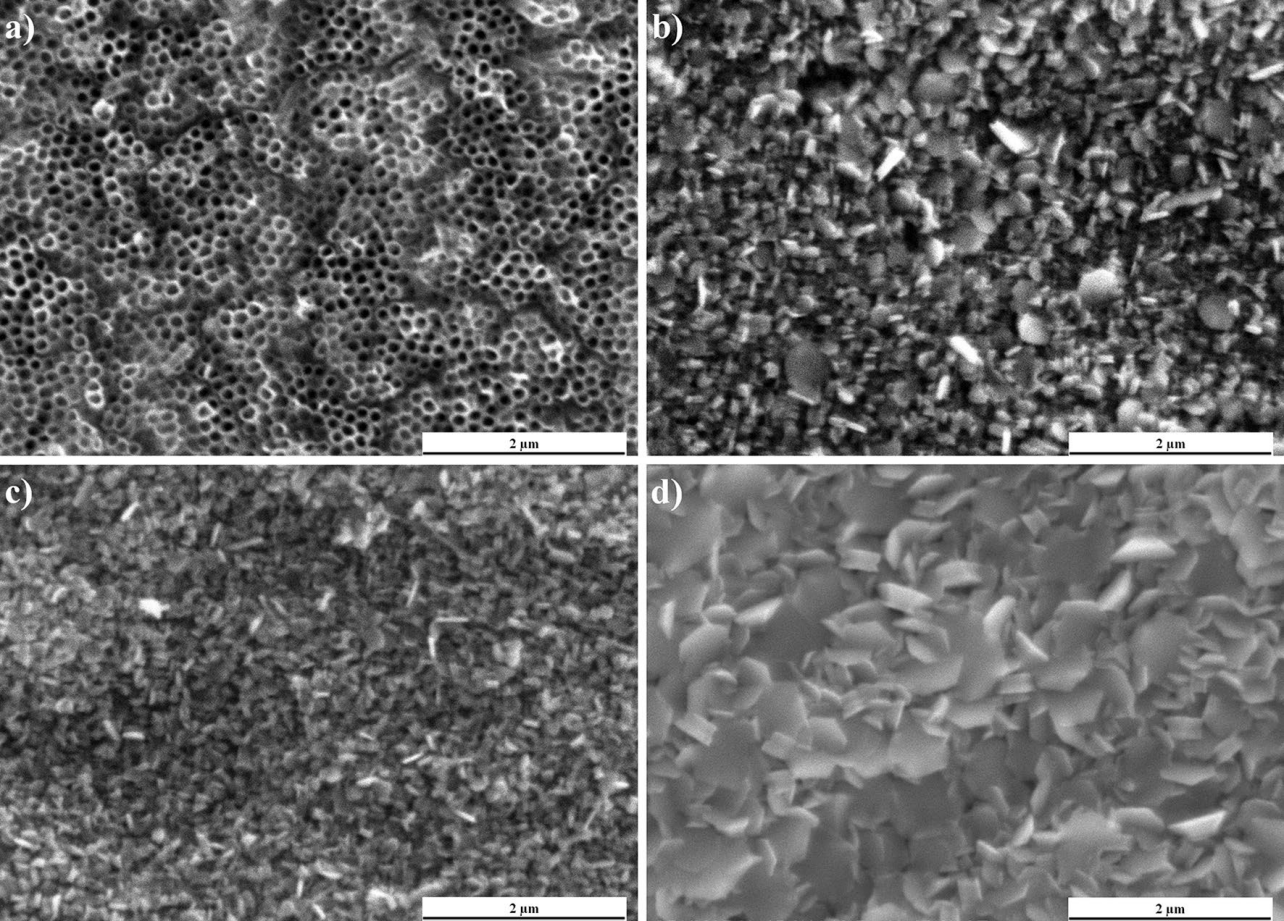
Surface SEM images: (**a**) bare nanotube surface, (**b**) Zn-1 nm, (**c**) Zn-3 nm and (**d**) Zn-5 nm.

The morphologies of nanotube and Zn-deposited nanotube surfaces of different thicknesses are given in Fig. 2. There are three stages (I, II, III) for the growth process of TiO_2_ nanotubes in the direction of the oxygen bubble model. These are [(i) the formation of oxide layer, (ii) tube initiation, and (iii) well-ordered nanotube growth] are better explained^1^. The ionic current promotes oxide growth. Subsequently, the electronic current produced by oxygen evolution maintains the nanotube growth. The oxide compact layer and the expansion of oxygen bubbles entrapped between electrolyte/oxide interface lead to a deformation of the compact oxide layer. This resulted in forming a hemispherical bottom that acts as a mold for the nanotubes. After the releasing of O_2_ gas and oxide layer growth rates are stable, self-organized nanotube growth (stage III) occurs^53,54^.

Homogeneous depositions were observed on the Zn-deposited surfaces and the nanotube morphology was completely covered by newly deposited Zn layers as seen in Fig. 2b–d. As a result, the surface chemistry and nanotube surface morphology have changed. Although the pore structure was partially preserved on the surface, the presence of nanotubes could not be preserved. This is due to the difference in the nature of the Zn structure observed during the TB process. The powder Zn metal rapidly adheres to the nanotube surface by spraying directly into the boiler within a short time, without showing melting behavior in the tungsten melting pot at low current values and for long periods. This situation causes morphological changes and laminar growths on nanotube surfaces as seen in Fig. 2. Depending on the increase in the deposition area on the nanotube surfaces, a trend from needle-like morphology to laminar morphology is observed with increasing thickness. Thus, the nanotube morphology is eliminated.

### EDX analysis of the surfaces

**Figure 3:**
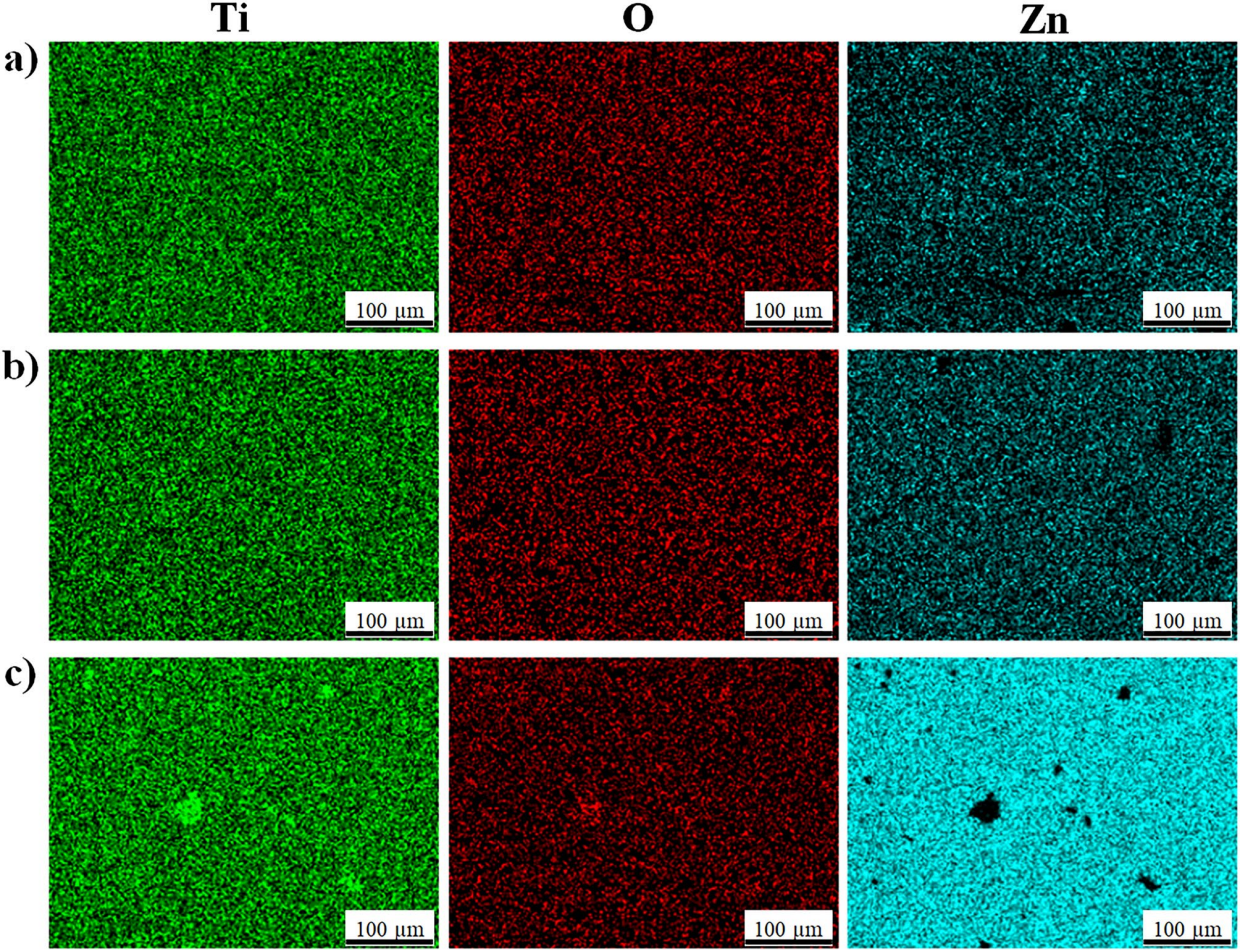
EDX-mapping images of Zn-deposited nanotube surfaces: (**a**) Zn-1 nm, (**b**) Zn-3 nm and (**c**) Zn-5 nm.

The Ti and O structures that homogeneously distributed throughout the surface were observed in all Zn-deposited samples as seen in Fig. 3. Also, as expected, Zn structures were also observed and homogeneous distribution was detected along the surface due to thermal deposition. However, the Zn signal could be relatively weak for Zn-1 nm and Zn-3 nm layers since e-beam with a 15 kV can penetrate to a depth to 1 µm. In addition, the amounts of Zn-based elements observed on nanotube surfaces are given in the Table 2. During the TB process, depending on the thickness values measured with the crystal thickness monitor integrated into the device, it was determined that the amount of antibacterial Zn accumulated with increasing thickness increased and values below the cytotoxic limit were observed. But as seen in Zn morphology; In particular, a very high rate of Zn was detected in the 5 nm coating. The layer that completely covers the surface in the SEM images and the elemental values support each other. Especially for Zn, coatings below 3 nm can be preferred to preserve the morphology and reduce the cytotoxic limit value to lower levels.

**Table 2:** Elemental amounts of nanotube and Zn-deposited nanotube coatings on cp-Ti (at. %).

Thickness	Ti	O	Zn
Bare nanotube (0 nm)	30.95	69.05	–
Zn-1 nm	28.41	62.64	8.95
Zn-3 nm	28.60	55.63	15.77
Zn-5 nm	21.34	21.19	57.47

### XRD analysis of the surfaces

**Figure 4:**
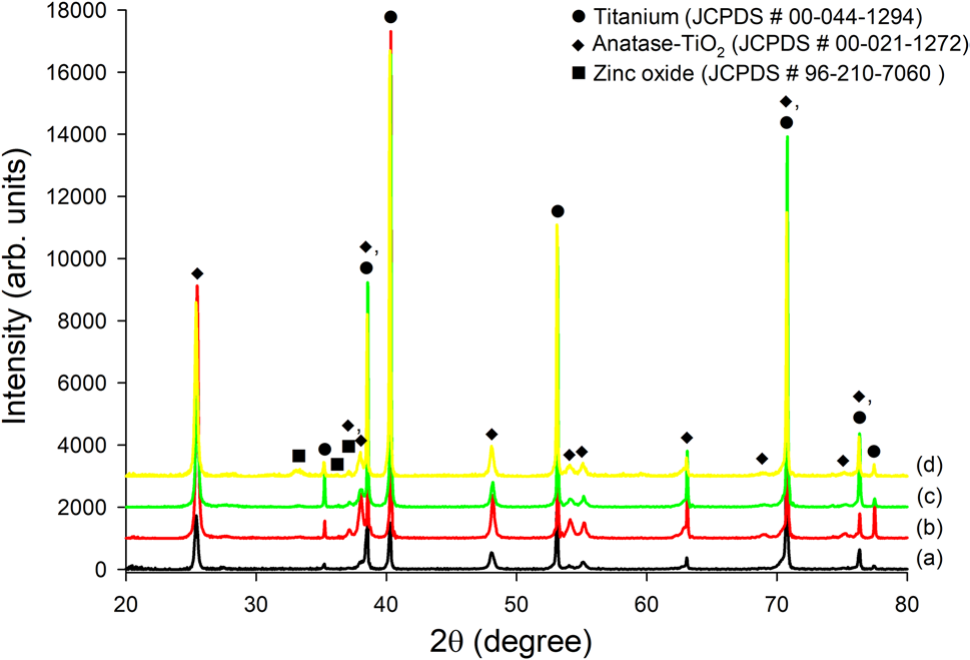
XRD spectra of bare nanotube and Zn-deposited nanotube surfaces: (**a**) bare nanotube, (**b**) Zn-1 nm, (**c**) Zn-3 nm and (**d**) Zn-5 nm.

Phase structures of bare nanotube and Zn-deposited nanotube surfaces of different thicknesses are illustrated in Fig. 4. The phases of Ti (titanium, JCPDS # 00-044-1294), anatase-TiO_2_ (anatase—titanium dioxide, JCPDS # 00-021-1272) and ZnO (zinc oxide, JCPDS # 96-210-7060) were observed in Zn-deposited nanotube surfaces while Ti (titanium, JCPDS # 00-044-1294), anatase-TiO_2_ (anatase—titanium dioxide, JCPDS # 00-021-1272) were detected in bare nanotube surfaces. The anodic oxidized nanotube surfaces are amorphous structures. After AO process, the nanotube surfaces are transformed from amorphous to crystalline structures without any morphological variations by a diffusion mechanism through 60 min at 450 °C as described in experimental section. In our previous works^48,55,56^, the AO formation mechanisms under the existence of F^-^ were discussed in detail. The AO process is the electrolysis of H_2_O at initial reactions. Subsequently, a compact TiO_2_ layer is occurred by dissolving of F^−^ on substrates. Finally, nano-pits occur at the last reaction transform into nanotube arrays at the suitable voltage, current and time during AO. Furthermore, as seen in Fig. 4b–d, a trace amount of ZnO was detected on the Zn-deposited nanotube surfaces. Already, the existence of elemental Zn structures was verified on all surfaces by EDX-mapping and EDX-area. The Zn powders were evaporated onto the nanotube surfaces under vacuum chamber through PVD-TE process. However, Zn-based nanotube surfaces can be transformed from metallic to ceramic form due to oxygen affinity of zinc under atmospheric conditions at post-fabrication PVD-TE process as shown in Fig. 4. Thus, the presence of bactericidal ZnO on the nanotube surfaces was confirmed by XRD.

### Roughness and topography of the surfaces

**Figure 5:**
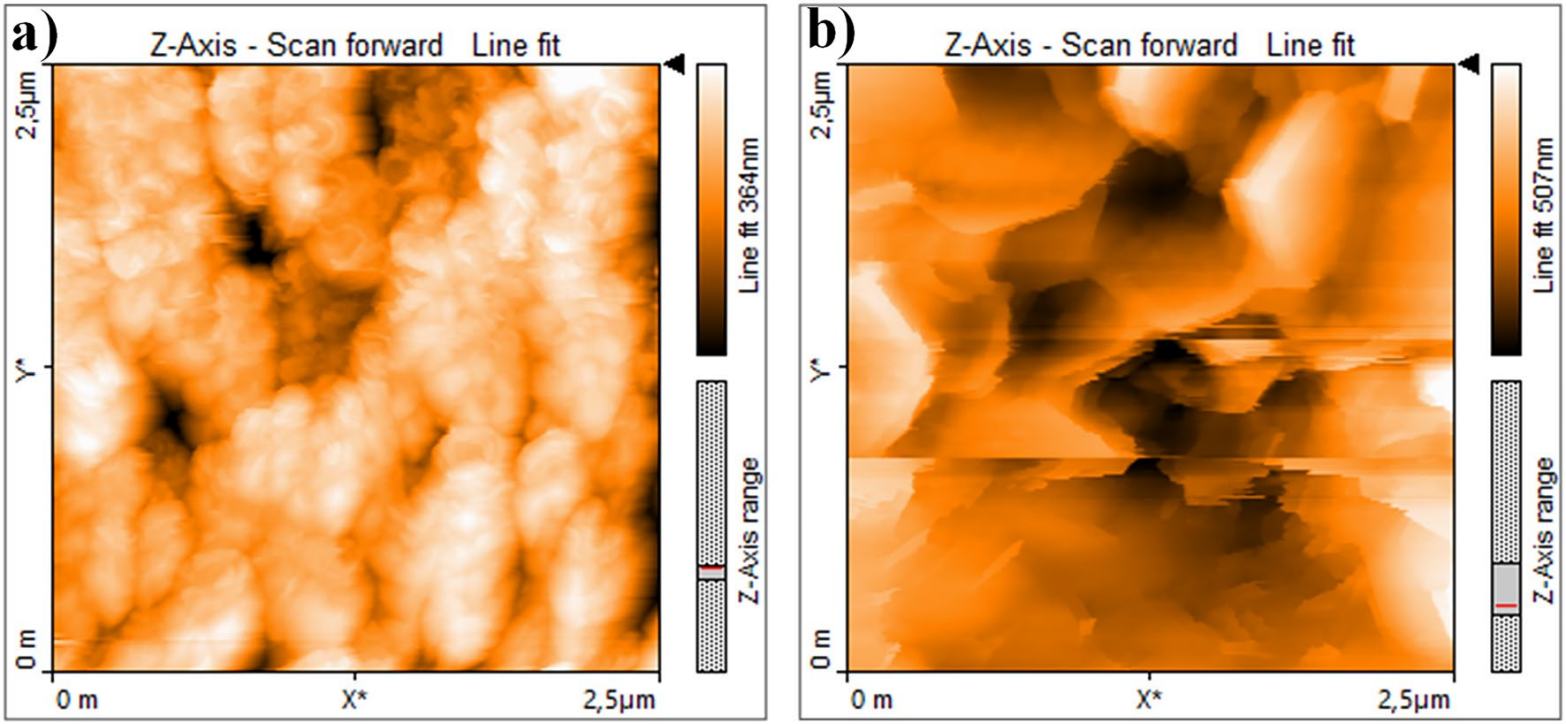
AFM images: (**a**) bare nanotube and (**b**) Zn-deposited nanotube surfaces.

Average roughness (Sa) results of bare cp-Ti, bare nanotube and Zn-deposited nanotube surfaces are given in Table 3. As can be seen from Table 3, the lowest roughness result was measured at 10.23 nm in the bare cp-Ti. Average roughness of bare nanotube surfaces is higher than one of bare cp-Ti due to existence of hollow nanotube morphology. Average roughness values of Zn-deposited nanotube surfaces; significantly increased compared to bare cp-Ti and bare nanotube surfaces. Compared to cp-Ti, the increase in surface chemistry as well as roughness values after AO and TB processes is one of the parameters that positively affect cell adhesion and tissue growth. The average surface roughness values of the Zn-deposited nanolayer coating structures vary between approximately 144–186 nm, respectively. However, average roughness of Zn-1 nm is great compared to Zn-3 nm whereas average roughness of Zn-5 nm is maximum value. These were also supported by surface SEM morphologies as seen in Fig. 2b–d. The surface morphologies on Zn-1 nm, Zn-3 nm and Zn-5 nm refer to lath, needle and planar surface structures, respectively. A bit more rough lath protruding structures appear on Fig. 2b compared to Fig. 2c due to deposition nature of Zn structures through PVD-TE process. This leads to reduce roughness from Zn-1 nm to Zn-3 nm as given in Table 3 since protruding structures on Zn-1 nm are transformed to thin needle-like low projection structures on Zn-3 nm. Moreover, more rough planar protruding structures appear on Fig. 2d compared to Fig. 2b,c due to deposition nature of Zn structures through PVD-TE process. This leads to increase roughness from Zn-3 nm to Zn-5 nm as given in Table 3 since thin needle-like low projection structures on Zn-3 nm are transformed to planar protruding structures on Zn-5 nm. As seen in Fig. 5, the AFM topographic images of the Zn-deposited nanotube surfaces support the laminar formation as seen in the SEM images.

**Table 3:** Average roughness (Sa) and tube length of bare cp-Ti, nanotube and Zn-deposited nanotube surfaces.

Thickness	Sa (nm)	Tube length (µm)
Bare cp-Ti	10.67	–
Bare nanotube	53.15	1.93
Zn-1 nm	160.09	–
Zn-3 nm	144.21	–
Zn-5 nm	185.72	–

### Wetting abilities of the surfaces

**Figure 6:**
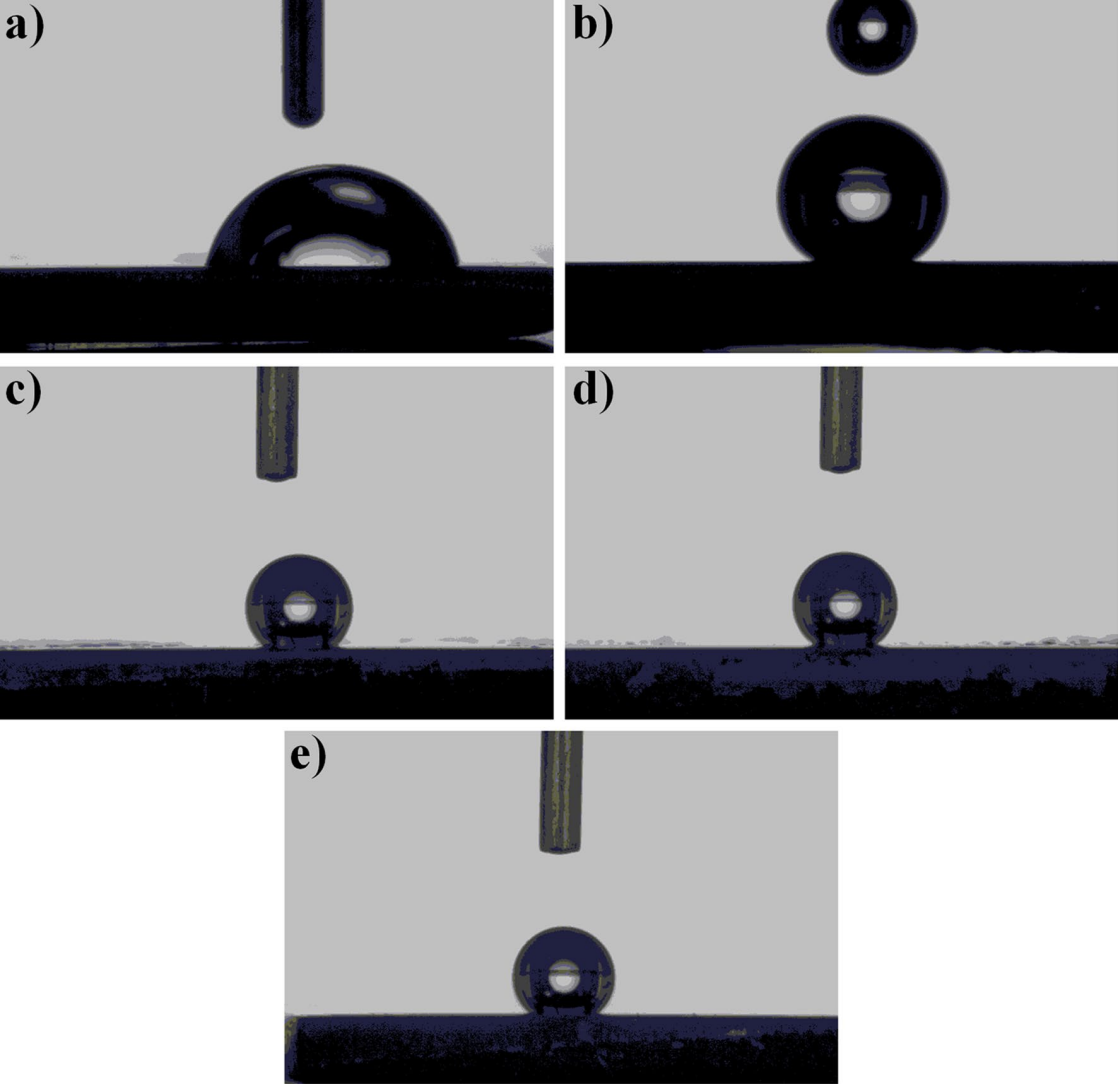
Contact angle measurement images: (**a**) bare cp-Ti, (**b**) bare nanotube, (**c**) Zn-1 nm, (**d**) Zn-3 nm and (**e**) Zn-5 nm.

Mean contact angle measurement results and wetting images of bare cp-Ti and Zn-deposited nanotube surfaces are given in Table 4 and Fig. 6, respectively. Surfaces with low contact angle show hydrophilic character as they can be completely wetted. Surfaces with a high contact angle exhibit hydrophobic character. While the contact angle of the surface is 90° and below, it shows hydrophilic property, while above 90° it shows hydrophobic property. The mean contact angle of the bare cp-Ti was measured as 79.7° ± 0.3. Therefore, cp-Ti shows hydrophilic properties. The mean contact angle measurement values of the Zn-deposited nanolayer coating structures on cp-Ti ranged from approximately 131.3° ± 0.1 to 149.7° ± 0.0, respectively. Bare cp-Ti surfaces generally showed lower wetting angle values than coated surfaces.

**Table 4:** Average contact angles of bare cp-Ti, bare nanotube and Zn-deposited nanotube surfaces.

Sample	Average contact angles (°)
Bare cp-Ti	79.2° ± 0.2
Bare nanotube	140.2° ± 0.2
Zn-1 nm	136.1° ± 0.1
Zn-3 nm	140.7° ± 0.0
Zn-5 nm	135.9° ± 0.1

The wettability of the surfaces depends on the surface energy, roughness, porosity and surface charges. The water droplet first comes into contact with the passive oxide surface on the bare cp-Ti surface, and the surface exhibits hydrophilic character due to the hydroxyl groups. TiO_2_ nanotube arrays generally exhibit hydrophilic or superhydrophilic properties as reported in the literature. The possible reasons could be that the enlarged gaps with more space between nanotubes stimulate liquid penetration or anatase causes hydrophilic properties^57^. This situation refers to the classical Wenzel model^58^. Wenzel model assumes that a water droplet fills up a rough surface. A completely wetted occurs, which depends on surface free energy roughness. The Wenzel model proposed a classical situation of a water droplet on a ‘flat’ surface by roughness. Eventually, a hydrophilic or superhydrophilic surface is occurred with great surface energy combines with nanoscale roughness. However, just like in this work contrary to the literature, a few opposite results have been reported in some studies. Yang et al. observed hydrophobic behavior and they increased contact angles from 77° to 141° under atmospheric air conditions at post-annealing of TiO_2_ nanotubes^59^. This wetting behavior can be ascribed to the replacement of the chemisorbed OH^−^ groups with O_2_ gaseous^60^ and the adsorption of organic contaminants on the TiO_2_ nanotube layer in atmospheric air conditions^59^. Therefore, contact angle values of TiO_2_ nanotube arrays are higher than substrate and literature works^59,61,62^. This type of result on TiO_2_ nanotube arrays was also observed in our previous study and discussed in detail^56^.

Similarly, the water drop touches the Zn nanolayer on the TiO_2_ nanotube surface. Although these surfaces are based on nanolayers, the surface chemistry and surface energy have also changed due to the antibacterial structures on the surface. For this reason, just like in AO-based nanotubes, the atmosphere gases inside the tubes create resistance for a short time in the first place and avoid the surface from getting wet. This result points to the Cassie–Baxter situation. According to this; the liquid occur a contact line with the air trapped below the contact line on the rough surface. In addition, Zn-based structures increase the hydrophobic properties of the surface by reducing the polar properties of the surface. This is supported by experimental results. Also, regardless of the nanotube morphology, the Zn-based nanolayer surface structure covered the nanotube surfaces, showing morphology ranging from needle-like to laminar surface structures. The Zn-based nanolayer showed mostly hydrophobic properties on the surfaces. Therefore, besides the surface morphology, the chemistry of the surface; it significantly affects the hydrophobic/hydrophilic behaviors of the surface. The hydrophilic/hydrophobic surface affects adhesion and spreading of the cells. While some studies in the literature indicate that cell proliferation is easy on hydrophilic surfaces, some studies have shown that cell adhesion is better on hydrophobic surfaces^63,64^. Moreover, it has been reported in the literature that hydrophobic surfaces absorb proteins more strongly than hydrophilic surfaces^65,66^. In addition, the hydrophobicity of the surface reduces bacterial adhesion and spread. Thus, it contributes antibacterial properties.

### Electrochemical corrosion properties of the surfaces

Open circuit potentials (OCP) and Tafel extrapolation results (corrosion current density (i_cor_), corrosion potential (E_cor_) and polarization resistance (Rp)) of bare cp-Ti and Zn-deposited nanotube surfaces are given in Table 5. Corrosion resistance depends on many parameters such as surface morphology, coating thickness, structural defects and phase structure. Bare cp-Ti metal showed lower corrosion current density by passivation at higher potential values compared to Zn-deposited nanotube surfaces. Thus, bare cp-Ti exhibited higher corrosion resistance. Here, the passive oxide layer on the bare cp-Ti surface causes passivation behavior. Predominantly oxide nanotube structures on the surfaces, the protective oxide layer on the surface creates a barrier between the substrate metal and the atmosphere, reducing the ion release. Moreover, metal nanolayers on the surface are hydrophobic, resulting in increased corrosion resistance. However, due to the biodegradable behavior of Zn on the nanotube surface, Zn-deposited nanotube surfaces showed higher corrosion current density than the bare metal. It has been concluded that the corrosion current density increases and corrosion resistance decreases due to the increase in the amount of Zn with increasing thickness of the Zn nanolayer structure. As a result; although the amount increases with increasing thickness with PVD-TE on nanotube surfaces, the solubility and hydrophilic/hydrophobic properties of the layers dominate the corrosion resistance. For implant applications, the antibacterial Zn structure on the nanotube surface must maintain its presence on the surface until the healing is completed, and then it is necessary for its gradual removal from the surface.

**Table 5:** Electrochemical corrosion parameters of bare cp-Ti and Zn-deposited nanotube surfaces.

	OCP (V)	E_cor_ (V)	I_cor_ (A/cm^2^)	Rp (ohm)
Bare cp-Ti	− 0.460	− 0.672	2.57 × 10^–6^	6160.4
Zn-1 nm	− 0.182	− 0.373	3.57 × 10^–6^	3837.1
Zn-3 nm	− 0.892	− 1.058	2.06 × 10^–5^	465.33
Zn-5 nm	− 0.941	− 1.061	5.85 × 10^–5^	255.46

### Antibacterial activities of the surfaces

**Figure 7:**
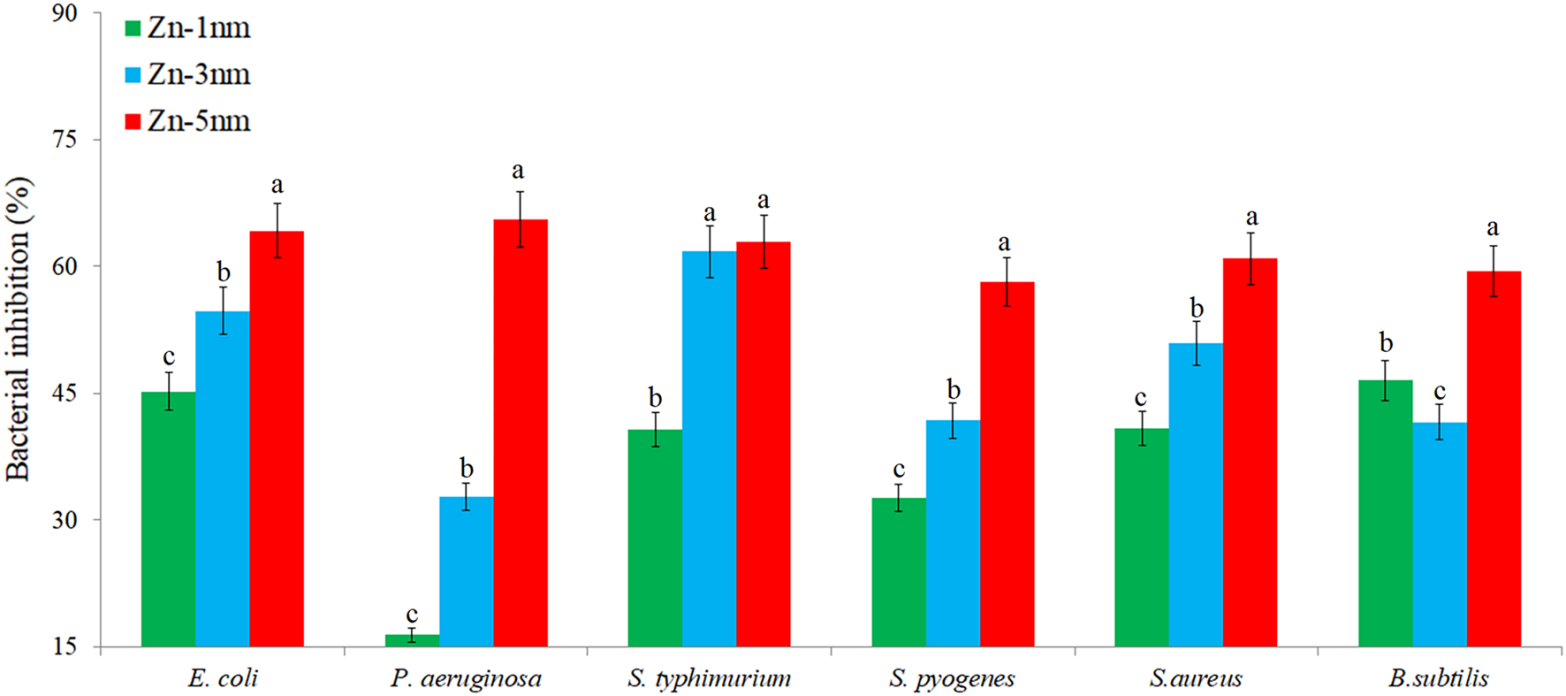
Percentage of microbial inhibition of Zn-deposited nanotube surfaces for Gram-positive and Gram-negative bacteria. Values shown with different letters for each bacteria are statistically significant.

**Figure 8:**
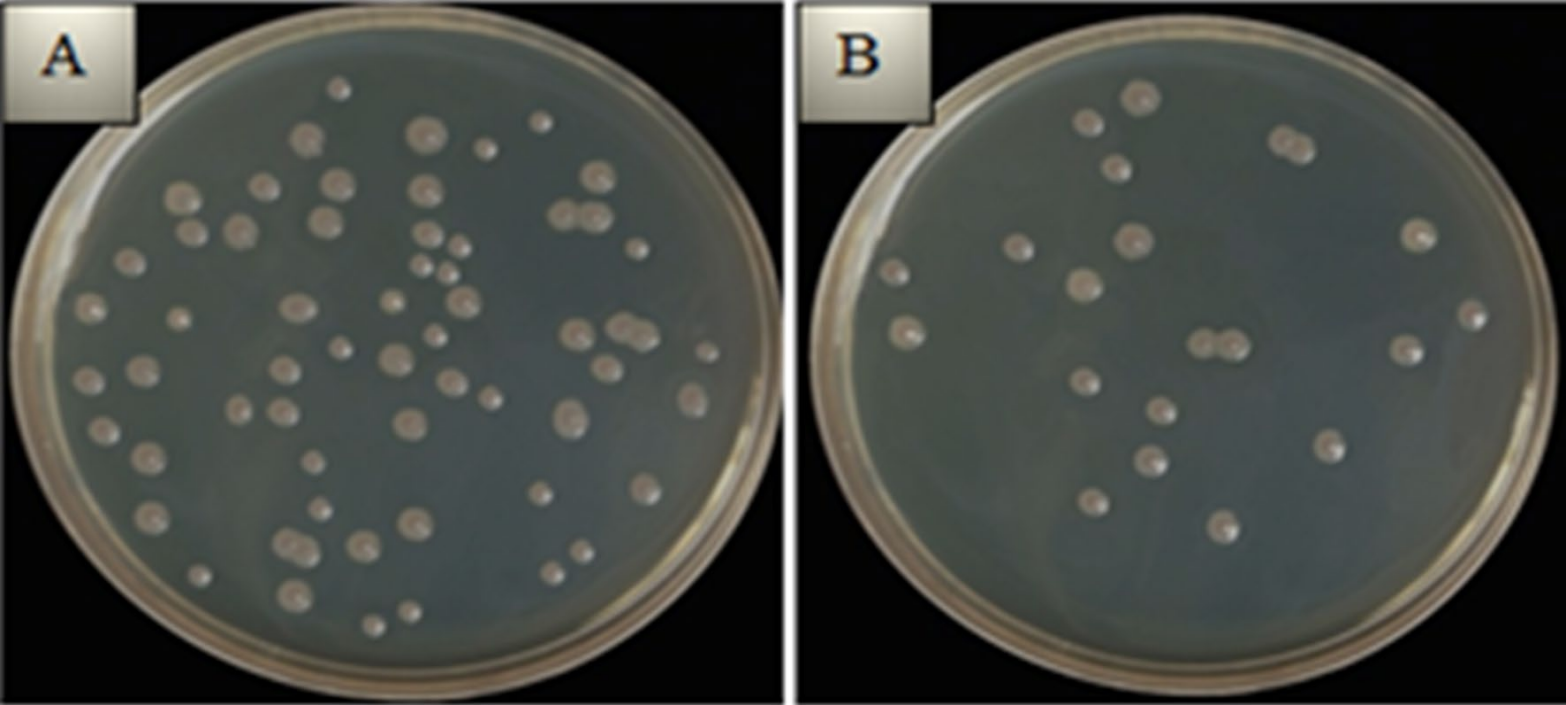
Decrease in bacterial colonies after recultivation in samples with the highest antibacterial activity on Zn-deposited surfaces: (**a**) *P. aeruginosa* viability after reculturation on bare cp-Ti surfaces, (**b**) *P. aeruginosa* viability after reculture on Zn-5 nm surfaces.

The cidal effect rates of Zn-deposited nanotube surfaces on different bacteria are given in the Fig. 7. Zn accumulation on nanotube surfaces caused a decrease in the number of colonies in bacteria. The highest antibacterial activity for Zn-deposited nanotube surfaces was found to be 62.9–66.6% in Gram-negative bacteria. When all Zn-deposited nanotube surfaces were examined, the highest bacterial inhibition was obtained with 5 nm surface against *P. aeruginosa* as seen in Fig. 8. In antibacterial studies with 3 nm surface, the highest inhibition was obtained against *S. typhimurium*, while 1 nm surface showed highest effect against *E. coli*. A lower inhibition was observed in gram positives and the highest inhibition among gram positives was obtained against *S. aureus* at 60%. This result shows that gram negatives are more sensitive to Zn-deposited nanotube surfaces. This result can be explained by the structural differences between gram positive and gram negative. Chemical modification of phosphatidylglycerol in gram-positive bacteria by bacterial enzymes is one of the main mechanisms by which bacteria develop resistance to many agents, especially cationic antimicrobials.

It has been concluded that the bacterial inhibition increases with the increase in the amount of Zn with increasing thickness of the Zn nanolayer structure. Observation of inhibition in all tested gram positive and gram negative bacteria indicates the broad spectrum effect of Zn-deposited nanotube surfaces. The inhibitory effect of Zn-deposited nanotube surfaces on bacteria can be explained by the antimicrobial activity of Zn ions. The electrostatic interaction between the positively charged Zn ions and the negatively charged bacterial cell wall may lead to disruptions in membrane integrity, deformation of transport molecules in the membrane, and deterioration in membrane permeability. Cell membrane damage after interaction with Zn-containing surfaces causes leakage of cytoplasm and a bacteriocidal effect occurs^67^. It is reported in the literature that Zn-deposited surfaces cause a selective inhibition. Pietrzyk et al. investigated the antibacterial effects of Zn-deposited surfaces against *E. coli.* They stated that antimicrobial activity improved with increasing Zn coating thickness. And, this resulted in a decreasing in *E. coli* colonization^68^. Hu et al. reported that Zn-deposited TiO_2_ surfaces exhibit a good ability to inhibit both gram-negative and gram-positive bacteria^39^.

### Cell viabilities of the surfaces

**Figure 9:**
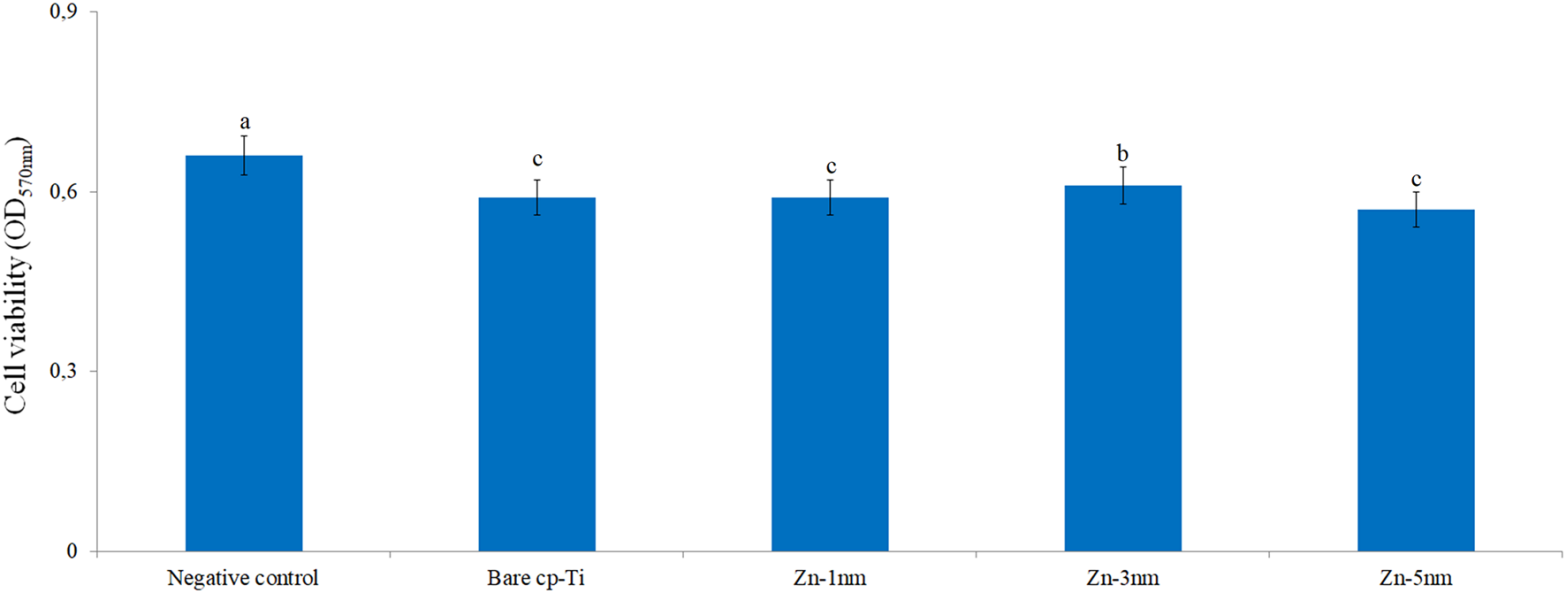
MTT test results of the bare cp-Ti and Zn-deposited nanotube surfaces after 4 h of incubation. Values shown with different letters are statistically significant.

The change in viability of cells adhering to bare cp-Ti and Zn-deposited nanotube surfaces was investigated by MTT test. It was determined that the viability of cells with adhesion on cp-Ti surfaces was generally decreased compared to the negative control group. Similarly, Kara and Pürçek determined that the viability of the SAOS-2 cell line decreased after interaction with Cp-Ti and Ti-6Al-4 V surfaces^69^. The change in the viability of cells adhering to Gr2 and Zn-deposited nanotube surfaces is given in Fig. 9. It was determined that cell viability was decreased on all Zn-deposited titanium surfaces compared to the negative control. However, it was determined that there was an increase in cell viability on Zn-deposited nanotube surfaces when compared to bare cp-Ti surfaces. This result indicates that titanium deposited Zn on the surface positively affects cell proliferation. As the thickness of Zn deposited on the surface changed, low-level and statistically insignificant changes were detected in cell viability. These changes are due to the effect of nanolayer structures formed after Zn deposition on the surface, affecting cell adhesion. In addition, increasing the Zn layer thickness (from 1 nm to 3) nm on nanotube surface positively affected cell viability. In the literature, Zhao et al. stated that the adhesion, proliferation and viability of osteoblast cells on Zn-deposited rough titanium surfaces are quite high and this situation is related to the nanoparticle surface. They also reported that the surfaces exhibited high antibacterial properties^70^. Contrary to these findings, Ortiz et al. observed a decrease in the viability of cells adhering to Zn apatite coated titanium surfaces compared to the titanium surface^71^.

### ALP activities of the surfaces

**Figure 10:**
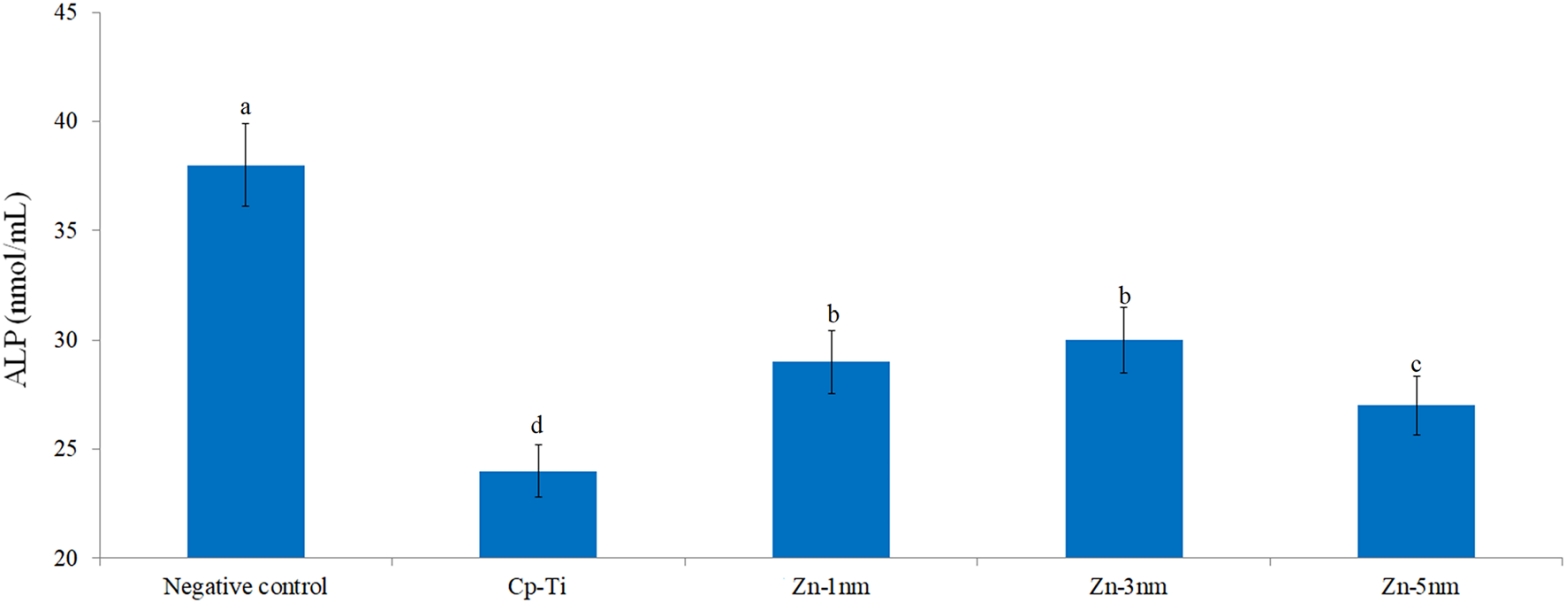
ALP activity in cells adhering to bare cp-Ti and Zn-deposited nanotube surfaces. Values shown with different letters are statistically significant.

A profile of ALP activity was obtained parallel to the cell viability data on Zn-deposited nanotube surfaces as shown in Fig. 10. Compared to the negative control, ALP activity was decreased on Zn-deposited nanotube surfaces. When the Zn-deposited nanotube surfaces are compared within themselves, it can be stated that the ALP activity increases depending on the Zn density on the surface. It is known that Zn metal increases protein synthesis and ALP activity in cells, and these increases positively affect cell viability^72,73^. Also, Yang et al. found that ALP activity increased for 14 days in osteoblast cells on Zn-deposited modified titanium surfaces^74^.

### Artificial blood proteins adsorptions of the surfaces

Protein adsorption on bare cp-Ti and Zn-deposited nanotube surfaces was studied in single protein solution mixtures in batch system and the results are given in Table 6. The protein adsorption tendency of the surfaces was investigated with albumin, globulin and fibrinogen proteins, which are among the major proteins in the blood. In general, a decrease in protein adsorption was observed after Zn coating for all tested proteins. Adsorption of albumin, fibrinogen and globulin to bare cp-Ti surfaces were 19.21 ± 0.53 mg/cm^2^, 17.72 ± 0.85 mg/cm^2^ and 14.91 ± 0.29 mg/cm^2^, respectively. After coating the nanotube surfaces with Zn, it was determined that the adsorption of all tested proteins decreased; the lowest adsorption for albumin and fibrinogen was obtained with Zn-3 nm, and for globulin with Zn-5 nm surfaces. However, no statistically significant difference was observed in globulin adsorption on Zn-5 nm and Zn-3 nm surfaces (p > 0.05). No direct correlation was found between protein adsorption and surface-deposited Zn density on all Zn-deposited nanotube surfaces tested.

**Table 6:** Protein adsorption of bare cp-Ti and Zn-deposited nanotube surfaces.

Sample	Albumin (mg/cm^2^)	Fibrinogen (mg/cm^2^)	Gamma-globulin (mg/cm^2^)
Bare cp-Ti	19.21 ± 0.53^a^	17.72 ± 0.85^a^	14.91 ± 0.29^a^
Zn-1 nm	12.17 ± 0.63^d^	12.94 ± 0.81^c^	11.51 ± 0.46^b^
Zn-3 nm	10.55 ± 0.71^e^	10.88 ± 0.64^d^	8.87 ± 0.21^c^
Zn-5 nm	13.92 ± 0.99^c^	11.29 ± 0.26^ cd^	8.32 ± 0.19^c^

Values are shown as mean ± SD. Means shown with different letters in the same column are statistically significant (p < 0.05).

Protein adsorption is closely related to the wettability and roughness of the surface. In addition, surface porosity also plays an important role in protein adsorption by creating a large adsorption area. Increases in surface roughness with surface modification methods cause decreases in protein adsorption^75,76^. Ferraris and Spriano reported that nanotubular structures with high roughness increase protein adsorption, and surfaces with high Zn reduce protein adsorption due to their lower roughness^77^. The albumin, fibrinogen and globulin adhesion test was investigated for blood compatibility. Adhesion of these proteins to the coating surfaces will also trigger the adhesion of blood cells. Adhesion of blood proteins to the surfaces of implants in contact with blood leads to numerous adverse reactions such as coagulation. Low adhesion of blood proteins is an important indicator of hemocompatibility. For this reason, the adhesion of blood proteins to the surface must be tested in the evaluation of blood compatibility.

**Figure 11:**
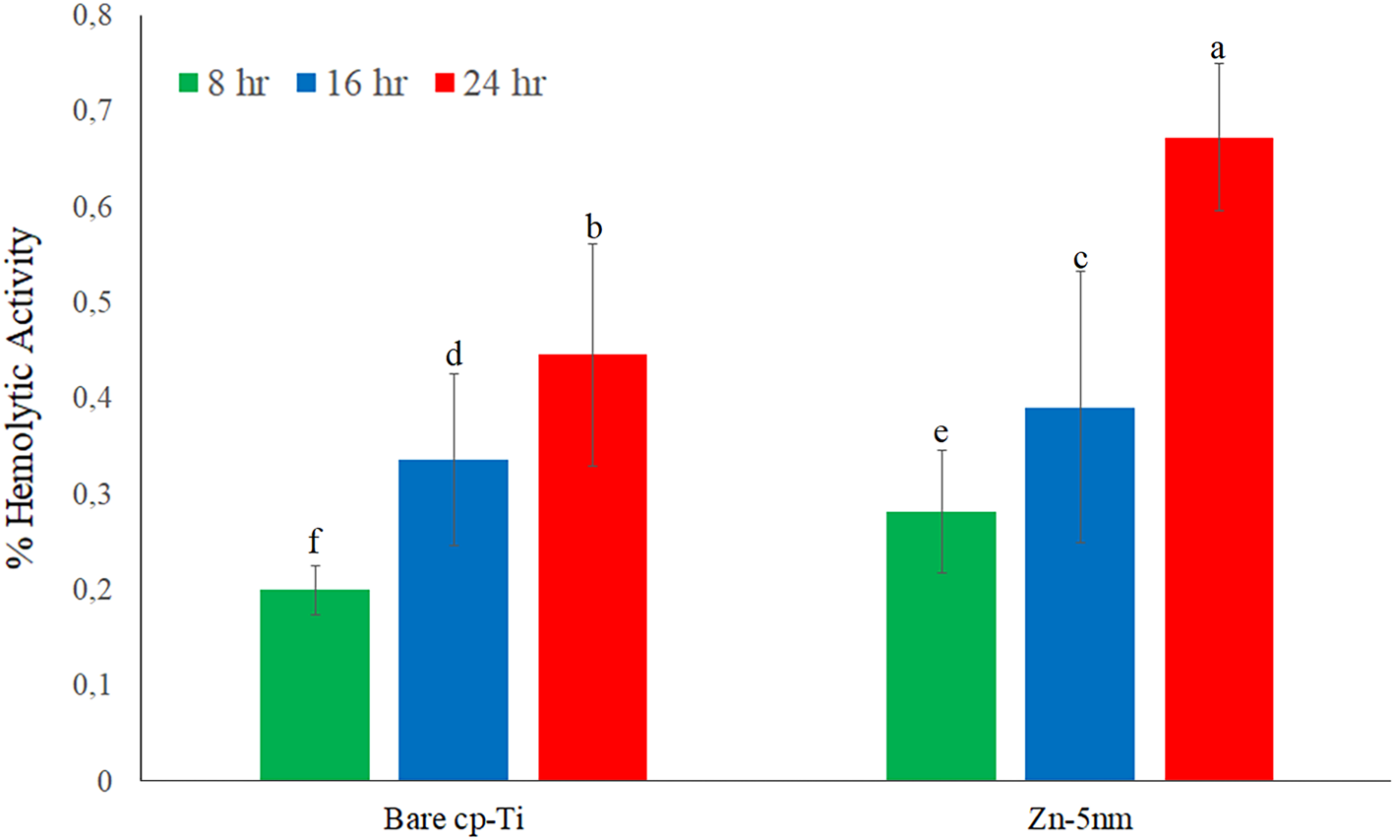
Hemolytic activities of bare cp-Ti and Zn-deposited (Zn-5 nm) nanotube surfaces for 8 h, 16 h and 24 h. Values shown with different letters are statistically significant.

Hemolytic activities of bare cp-Ti and Zn-deposited nanotube surfaces for different periods are given in the Fig. 11. In general, it was determined that the hemolytic activity increased with the increase in time. It was determined that the hemolytic activity after Zn coating processes increased compared to bare cp-Ti surfaces. Bare cp-Ti surfaces exhibited 0.2–0.44% hemolytic activity. The Zn-deposited nanotube surfaces tested between 8, 16 and 24 h showed hemolytic activity in the range of 2.28–0.67%.

Despite these increases observed in hemolytic activity, both bare cp-Ti and Zn-deposited nanotube surfaces are acceptable as surfaces that do not exhibit hemolytic activity. In the literature, biomaterials are classified in three different categories depending on hemolysis: Materials resulting in hemolysis above 5% are classified as hemolytic, between 5 and 2% as mild hemolytic, and below 2% as non-hemolytic^78^. According to this classification, the surfaces obtained by Zn coating processes did not exhibit hemolytic activity and preserved their blood compatible feature.

## Conclusions

The present study evaluated the Zn-deposited nanotube surfaces on cp-Ti surfaces using the AO and PVD-TE processes with positive results for biomedical implant applications. The use of the AO and PVD-TE processes induce the formation of homogenous and high purity layers and have the aim of improving antibacterial and biocompatible properties of the dental and orthopedic implants. After Zn-deposition, Zn layers were homogenously covered onto nanotubes. Ti, O and Zn observed on the surfaces were distributed homogenously through the whole surface. Ti, anatase-TiO_2_ and bactericidal ZnO phases were obtained on Zn-deposited nanotube surfaces. Zn-deposited nanotube and bare nanotube surfaces exhibited hydrophobic character while bare cp-Ti indicated hydrophilic properties. This is beneficial for antibacterial activities as supported in results. Zn-deposited nanotube surfaces showed higher corrosion current density than the bare metal under SBF conditions. Cell viability, ALP activity and antibacterial properties of Zn-deposited nanotube surfaces were improved with compared to bare cp-Ti. In addition, hemolytic activity and protein adsorption of Zn-deposited nanotube surfaces were reduced. All of these preliminary results suggest a good interaction with living tissue. Thus, the Zn-deposited TiO_2_ nanotube coatings of titanium studied in this work offer very promising results for future dental implant applications due to the properties of antibacterial and biocompatible.

## Data Availability

The datasets used and/or analyzed during the current study are available from the corresponding author on reasonable request.

## References

[1] Rios J (2022). Self-organized TiO_2_ nanotubes on Ti–Nb–Fe alloys for biomedical applications: Synthesis and characterization. Electrochem. Commun..

[2] Kulkarni M (2015). Titanium nanostructures for biomedical applications. Nanotechnology.

[3] Niinomi M, Nakai M, Hieda J (2012). Development of new metallic alloys for biomedical applications. Acta Biomater..

[4] Jaafar A, Hecker C, Arki P, Joseph Y (2020). Sol–gel derived hydroxyapatite coatings for titanium implants: A review. Bioeng. Basel..

[5] Baltatu MS, Sandu AV, Nabialek M, Vizureanu P, Ciobanu G (2021). Biomimetic deposition of hydroxyapatite layer on titanium alloys. Micromachines.

[6] Kim J (2021). Characterization of titanium surface modification strategies for osseointegration enhancement. Metals..

[7] Sharkeev Y (2022). Development of ultrafine–grained and nanostructured bioinert alloys based on titanium, zirconium and niobium and their microstructure, mechanical and biological properties. Metals.

[8] Punset M (2021). Citric acid passivation of titanium dental implants for minimizing bacterial colonization impact. Coatings.

[9] Hameed HA (2022). Osteoblastic cell responses of copper nanoparticle coatings on Ti-6Al-7Nb alloy using electrophoretic deposition method. Biomed. Res. Int..

[10] García-Serrano J, Gómez-Hernández E, Ocampo-Fernández M, Pal U (2009). Effect of Ag doping on the crystallization and phase transition of TiO_2_ nanoparticles. Curr. Appl. Phys..

[11] Lee S-B, Otgonbayar U, Lee J-H, Kim K-M, Kim K-N (2010). Silver ion-exchanged sodium titanate and resulting effect on antibacterial efficacy. Surf. Coat. Technol..

[12] Song D-H (2012). Synthesis of titanium oxide thin films containing antibacterial silver nanoparticles by a reactive magnetron co-sputtering system for application in biomedical implants. Mater. Res. Bull..

[13] Zhang XX (2021). Enhanced uniformity, corrosion resistance and biological performance of Cu-incorporated TiO2 coating produced by ultrasound-auxiliary micro-arc oxidation. Appl. Surf. Sci..

[14] Demirbaş Ç, Ayday A (2021). Effect of Ag concentration on structure and wear behaviour of coatings formed by micro-arc oxidation on Ti6Al4V Alloy. Surf. Eng..

[15] Chernozem RV (2019). Functionalization of titania nanotubes with electrophoretically deposited silver and calcium phosphate nanoparticles: Structure, composition and antibacterial assay. Mater. Sci. Eng. C.

[16] Yang T, Qian S, Qiao Y, Liu X (2016). Cytocompatibility and antibacterial activity of titania nanotubes incorporated with gold nanoparticles. Colloids Surf. B.

[17] Khudhair D (2021). Investigation of effects of copper, zinc, and strontium doping on electrochemical properties of titania nanotube arrays for neural interface applications. Processes.

[18] Khudhair D (2016). Anodization parameters influencing the morphology and electrical properties of TiO_2_ nanotubes for living cell interfacing and investigations. Mater. Sci. Eng. C Mater. Biol. Appl..

[19] Ozkan S, Nguyen NT, Mazare A, Cerri I, Schmuki P (2016). Controlled spacing of self-organized anodic TiO_2_ nanotubes. Electrochem. Commun..

[20] Necula MG (2022). Macrophage-like cells are responsive to titania nanotube intertube;an in vitro study. Int. J. Mol. Sci..

[21] Cheng Y (2018). Progress in TiO_2_ nanotube coatings for biomedical applications: A review. J. Mater. Chem. B.

[22] Soares P, Dias-Netipanyj MF, Elifio-Esposito S, Leszczak V, Popat K (2018). Effects of calcium and phosphorus incorporation on the properties and bioactivity of TiO_2_ nanotubes. J. Biomater. Appl..

[23] Dias-Netipanyj MF (2020). Crystallinity of TiO_2_ nanotubes and its effects on fibroblast viability, adhesion, and proliferation. J. Mater. Sci. Mater. Med..

[24] Li L (2019). Nanotopography on titanium promotes osteogenesis via autophagy-mediated signaling between YAP and β-catenin. Acta Biomater..

[25] Alamdari AA (2022). In vitro antibacterial and cytotoxicity assessment of magnetron sputtered Ti1.5ZrTa0.5Nb0.5W0.5 refractory high-entropy alloy doped with Ag nanoparticles. Vacuum.

[26] Aydogan DT, Muhaffel F, Cimenoglu H (2022). Hydrothermal treatment of the silver-incorporated MAO coated Ti6Al7Nb alloy. Surf. Innov..

[27] Haugen HJ, Makhtari S, Ahmadi S, Hussain B (2022). The antibacterial and cytotoxic effects of silver nanoparticles coated titanium implants: A narrative review. Materials..

[28] Yada M (2017). Synthesis and antibacterial activity of a silver nanoparticle/silver titanium phosphate-nanocomposite nanobelt thin film formed on a titanium plate. Thin Solid Films.

[29] Zhang YX (2018). Enhanced silver loaded antibacterial titanium implant coating with novel hierarchical effect. J. Biomater. Appl..

[30] Zhang Y (2022). Microstructure and properties in simulated seawater of copper-doped micro-arc coatings on TC4 alloy. Coatings.

[31] Ghosh R, Swart O, Westgate S, Miller BL, Yates MZ (2019). Antibacterial copper-hydroxyapatite composite coatings via electrochemical synthesis. Langmuir.

[32] Hu YD (2022). Construction of mussel-inspired dopamine-Zn^2+^ coating on titanium oxide nanotubes to improve hemocompatibility, cytocompatibility, and antibacterial activity. Front. Bioeng. Biotechnol..

[33] Jin GD (2014). Osteogenic activity and antibacterial effect of zinc ion implanted titanium. Colloids Surf. B-Biointerfaces.

[34] Petrini P (2006). Antibacterial activity of zinc modified titanium oxide surface. Int. J. Artif. Organs.

[35] Zhang X (2016). Corrosion behavior of Zn-incorporated antibacterial TiO_2_ porous coating on titanium. Ceram. Int..

[36] Sopchenski L, Popat K, Soares P (2018). Bactericidal activity and cytotoxicity of a zinc doped PEO titanium coating. Thin Solid Films.

[37] Yamaguchi M (1998). Role of zinc in bone formation and bone resorption. J. Trace Elem. Exp. Med..

[38] O'Connor JP, Kanjilal D, Teitelbaum M, Lin SS, Cottrell JA (2020). Zinc as a therapeutic agent in bone regeneration. Materials..

[39] Hu H (2012). Antibacterial activity and increased bone marrow stem cell functions of Zn-incorporated TiO_2_ coatings on titanium. Acta Biomater..

[40] Zhao Q-M, Cheng L, Liu Z-T, Zhao J-J (2014). Surface characteristics of Zinc–TiO_2_ coatings prepared via micro-arc oxidation. Compos. Interfaces.

[41] Aydin EB, Siğircik G, Takci HAM (2021). Antimicrobial properties and corrosion behavior of TiO_2_NTs electrodes modified with Ag and ZnO nanorod in simulated body fluid solution. J. Mol. Struct..

[42] Vranceanu DM (2022). Electrochemical surface biofunctionalization of titanium through growth of TiO_2_ nanotubes and deposition of Zn doped hydroxyapatite. Coatings.

[43] Xiang Y (2018). Infection-prevention on Ti implants by controlled drug release from folic acid/ZnO quantum dots sealed titania nanotubes. Mater. Sci. Eng. C.

[44] Huo K (2013). Osteogenic activity and antibacterial effects on titanium surfaces modified with Zn-incorporated nanotube arrays. Biomaterials.

[45] Mohan L, Dennis C, Padmapriya N, Anandan C, Rajendran N (2020). Effect of electrolyte temperature and anodization time on formation of TiO_2_ nanotubes for biomedical applications. Mater. Today Commun..

[46] Roguska A, Pisarek M, Andrzejczuk M, Lewandowska M (2014). Synthesis and characterization of ZnO and Ag nanoparticle-loaded TiO_2_ nanotube composite layers intended for antibacterial coatings. Thin Solid Films.

[47] Durdu S, Cihan G, Yalcin E, Altinkok A (2021). Characterization and mechanical properties of TiO_2_ nanotubes formed on titanium by anodic oxidation. Ceram. Int..

[48] Durdu S (2021). Surface characterization of TiO_2_ nanotube arrays produced on Ti6Al4V alloy by anodic oxidation. Surf. Coat. Technol..

[49] Kokubo T, Takadama H (2006). How useful is SBF in predicting in vivo bone bioactivity?. Biomaterials.

[50] Durdu S (2022). Characterization and investigation of properties of copper nanoparticle coated TiO_2_ nanotube surfaces on Ti6Al4V alloy. Mater. Chem. Phys..

[51] Sharma P, Sharma JD (2001). In vitro hemolysis of human erythrocytes—by plant extracts with antiplasmodial activity. J. Ethnopharmacol..

[52] Aslam FRN, Riaz M, Zubair M, Rizwan K, Abbas M, Bukhari TH, Bukhari IH (2011). Antioxidant, haemolytic activities and GC-MS Profiling of *Carissa carandas* roots. Int. J. Phytomed..

[53] Li CY (2022). A review: Research progress on the formation mechanism of porous anodic oxides. Nanoscale Adv..

[54] Li P (2021). The effect of atmospheric pressure on the growth rate of TiO_2_ nanotubes: Evidence against the field-assisted dissolution theory. Electrochem. Commun..

[55] Nowruzi F, Imani R, Faghihi S (2022). Effect of electrochemical oxidation and drug loading on the antibacterial properties and cell biocompatibility of titanium substrates. Sci. Rep..

[56] Durdu S, Yalçin E, Altinkök A, Çavuşoğlu K (2023). Characterization and investigation of electrochemical and biological properties of antibacterial silver nanoparticle-deposited TiO_2_ nanotube array surfaces. Sci. Rep..

[57] Liu G, Du K, Wang K (2016). Surface wettability of TiO_2_ nanotube arrays prepared by electrochemical anodization. Appl. Surf. Sci..

[58] Wenzel RN (1936). Resistance of solid surfaces to wetting by water. Ind. Eng. Chem..

[59] Yang L (2014). Effect of annealing temperature on wettability of TiO_2_ nanotube array films. Nanoscale Res. Lett..

[60] Wang R (1998). Photogeneration of highly amphiphilic TiO2 surfaces. Adv. Mater..

[61] Sarraf M (2015). Effect of microstructural evolution on wettability and tribological behavior of TiO_2_ nanotubular arrays coated on Ti-6Al-4V. Ceram. Int..

[62] Shin DH, Shokuhfar T, Choi CK, Lee SH, Friedrich C (2011). Wettability changes of TiO_2_ nanotube surfaces. Nanotechnology.

[63] Faucheux N, Schweiss R, Lutzow K, Werner C, Groth T (2004). Self-assembled monolayers with different terminating groups as model substrates for cell adhesion studies. Biomaterials.

[64] Ikada Y (1994). Surface modification of polymers for medical applications. Biomaterials.

[65] Wang K, Zhou C, Hong Y, Zhang X (2012). A review of protein adsorption on bioceramics. Interface Focus.

[66] Vogler EA (2012). Protein adsorption in three dimensions. Biomaterials.

[67] Wang P, Zhang D, Qiu R, Hou B (2011). Super-hydrophobic film prepared on zinc as corrosion barrier. Corros. Sci..

[68] Pietrzyk B, Porebska K, Jakubowski W, Miszczak S (2019). Antibacterial properties of Zn doped hydrophobic SiO_2_ Coatings produced by sol–gel method. Coatings.

[69] Kara G, Purcek G (2020). Mechanical properties and cell proliferation response of borided biomedical titanium alloys with different crystalline structures. Surf. Coat. Technol..

[70] Zhao Q-M, Li G-Z, Zhu H-M, Cheng L (2017). Study on effects of titanium surface microporous coatings containing zinc on osteoblast adhesion and its antibacterial activity. Appl. Bionics Biomech..

[71] Ortiz IY (2016). In vitro assessment of zinc apatite coatings on titanium surfaces. Ceram. Int..

[72] Matsui T, Yamaguchi M (1995). Zinc modulation of insulin-like growth factor's effect in osteoblastic MC3T3-E1 cells. Peptides.

[73] Lutz W, Burritt MF, Nixon DE, Kao PC, Kumar R (2000). Zinc increases the activity of vitamin D-dependent promoters in osteoblasts. Biochem. Biophys. Res. Commun..

[74] Yang F (2012). Osteoblast response to porous titanium surfaces coated with zinc-substituted hydroxyapatite. Oral. Surg. Oral. Med. Oral. Pathol. Oral. Radiol..

[75] Rechendorff K, Hovgaard MB, Foss M, Zhdanov VP, Besenbacher F (2006). Enhancement of protein adsorption induced by surface roughness. Langmuir.

[76] Jimbo R, Ivarsson M, Koskela A, Sul YT, Johansson CB (2010). Protein adsorption to surface chemistry and crystal structure modification of titanium surfaces. J. Oral Maxillofac. Res..

[77] Ferraris S, Spriano S (2016). Antibacterial titanium surfaces for medical implants. Mater. Sci. Eng. C.

[78] Totea G, Ionita D, Demetrescu I, Mitache M (2014). In vitro hemocompatibility and corrosion behavior of new Zr-binary alloys in whole human blood. Open Chem..

